# An interdisciplinary framework for measuring and supporting adherence in HIV prevention trials of ARV-based vaginal rings

**DOI:** 10.7448/IAS.17.3.19158

**Published:** 2014-09-08

**Authors:** Kathleen M MacQueen, Elizabeth E Tolley, Derek H Owen, K Rivet Amico, Kathleen M Morrow, Thomas Moench, David R Friend, Barbara Friedland

**Affiliations:** 1 Social and Behavioral Health Sciences, FHI 360, Durham, NC, USA; 2 Contraceptive Technology Institute, FHI 360, Durham, NC, USA; 3 Department of Health Behavior and Health Education, School of Public Health, University of Michigan, Ann Arbor, MI, USA; 4 Centers for Behavioral & Preventive Medicine, The Miriam Hospital, Providence, RI, USA; 5 Department of Psychiatry & Human Behavior, Warren Alpert Medical School of Brown University, Providence, RI, USA; 6 Reprotect Inc, Baltimore, MD, USA; 7 The Johns Hopkins University, Baltimore, MD, USA; 8 Product Development, CONRAD, Arlington, VA, USA; 9 Population Council, New York, NY, USA

**Keywords:** vaginal ring, acceptability, adherence, clinical trial research, HIV prevention, measurement

## Abstract

**Introduction:**

Product adherence and its measurement have emerged as a critical challenge in the evaluation of new HIV prevention technologies. Long-acting ARV-based vaginal rings may simplify use instructions and require less user behaviour, thereby facilitating adherence. One ARV-based ring is in efficacy trials and others, including multipurpose rings, are in the pipeline. Participant motivations, counselling support and measurement challenges during ring trials must still be addressed. In previous HIV prevention trials, this has been done largely using descriptive and post-hoc methods that are highly variable and minimally evaluated. We outline an interdisciplinary framework for systematically investigating promising strategies to support product uptake and adherence, and to measure adherence in the context of randomized, blinded clinical trials.

**Discussion:**

The interdisciplinary framework highlights the dual use of adherence measurement (i.e. to provide feedback during trial implementation and to inform interpretation of trial findings) and underscores the complex pathways that connect measurement, adherence support and enacted adherence behaviour. Three inter-related approaches are highlighted: 1) adherence support – sequential efforts to define motivators of study product adherence and to develop, test, refine and evaluate adherence support messages; 2) self-reported psychometric measures – creation of valid and generalizable measures based in easily administered scales that capture vaginal ring use with improved predictive ability at screening, baseline and follow-up that better engage participants in reporting adherence; and 3) more objective measurement of adherence – real-time adherence monitoring and cumulative measurement to correlate adherence with overall product effectiveness through innovative designs, models and prototypes using electronic and biometric technologies to detect ring insertion and/or removal or expulsion. Coordinating research along these three pathways will result in a comprehensive approach to product adherence within clinical trials.

**Conclusions:**

Better measurement of adherence will not, by itself, ensure that future effectiveness trials will be able to address the most basic question: if the product is used per instructions, will it prevent HIV transmission? The challenges to adherence measurement must be addressed as one component of a more integrated system that has as its central focus adherence as a behaviour emerging from the social context of the user.

## Introduction

In July 2010, the CAPRISA 004 clinical trial of tenofovir 1% gel demonstrated that an ARV-based vaginal gel could prevent acquisition of HIV [1]. Subsequently, results from the iPrEX, Partners PrEP, TDF2 and Bangkok Tenofovir trials of oral ARV dosing further bolstered confidence in ARV-based prevention [2–5]. In each of these trials, sub-analyses indicated that poor adherence to the prescribed dosing regimen reduced efficacy. The importance of adherence to product use instructions was underscored by results from the VOICE and FEM-PrEP trials, where poor adherence to vaginal (VOICE) and oral (VOICE and FEM-PrEP) dosing regimens resulted in an inability to determine effectiveness [6, 7].

The delivery of microbicides through vaginal rings is perceived by some as a way to achieve better adherence, in part because rings have the potential to simplify the behavioural requirements for use. A number of long-acting ARV-based and multi-purpose prevention rings are in development. Two phase 3 clinical trials are now underway to assess the efficacy of a 28-day vaginal ring containing the antiretroviral drug dapivirine to reduce HIV transmission among African women [8]. Other rings in development include CONRAD's 90-day ring that contains both tenofovir and a contraceptive; the International Partnership for Microbicide's (IPM) dapivirine ring with or without a contraceptive added that would be effective for 30 days and potentially up to 60–90 days; the Population Council's 90-day ring containing MIV-150, zinc acetate and carrageenan, with or without a contraceptive, designed to protect against HIV, herpes simplex virus type-2 and human papillomavirus (Barbara Friedland, personal communication, 2014); and Merck's project to develop their ethylene vinyl acetate copolymer contraceptive ring as a method for also delivering ARVs. It is anticipated that over the next three years, multiple products will move from preclinical to clinical testing of safety and pharmacokinetics during use ranging from 30 days to 12 months.

Social science research with participants in trials of microbicides and PrEP indicate that a wide array of factors may potentially influence adherence. For example, lack of understanding about clinical research practices (e.g. how biological specimens will be used) [9], concerns about product safety [10] and distrust of the motivations of researchers and trial sponsors (e.g. motivated by negative values such as greed and racism) [9, 11, 12] are frequently described for HIV prevention research participants and communities. For women, decisions about whether and what to disclose to male partners about product use and research participation reflect culturally regulated gender dynamics that can either generate support or create obstacles for a woman's decision to participate in a trial and adhere to product use [10, 13–17]. There is also increasing recognition of the fact that the on-going and intensive level of interaction among participants in the research clinic waiting room and between participants and research staff foster the emergence of a research culture that may exacerbate or mitigate these other challenges [10].

Research conducted by IPM in preparation for phase 3 trials of the 30-day dapivirine ring found that women reported removal or expulsion of the ring between study visits at frequencies ranging from 4 to 18% of participants over varying time frames [18, 19]. The most common reasons cited for ring removal were menses, periodic cleaning and expulsion during urination or defecation. Currently, self-report is the primary method for estimating the number of hours or days that a removed or expelled ring remains outside the vagina before it (or a new ring) is inserted. Although self-reported adherence has been shown to over-estimate product use to varying degrees in ARV-based HIV prevention trials, collecting information on reasons for non-adherence and participants’ experiences with product use more generally is essential to providing effective counselling and support for proper use of all such products, including vaginal rings. Recognition of adherence challenges is necessary but not sufficient to ensure that participants receive adequate support for adherence at the level needed to evaluate product effectiveness. Also important are motivations for adherence within the context of a clinical trial. Most research on adherence outcomes has focused on the treatment context, where the goal is to maximize health benefits for clients. In the trial context, the health benefits of product use are unclear and may be non-existent, especially if there is randomization to a placebo arm. There is also greater ambiguity about the potential risks than is normally the case with approved medications. These unique clinical trial elements must be considered in optimizing clinical trial adherence to ring use. Additional consideration must be given to how the clinical trial elements are situated relative to the social context of the trial participants, for example, how the motivations of researchers are understood in the community.

Because vaginal rings can be inserted and removed by the user, direct observation of enacted adherence, that is, the actual act of using the product over time, is not feasible. In this regard, the adherence measurement challenges for rings are similar to those faced in identifying a valid, reliable and accurate measure of microbicide adherence with respect to other drug delivery systems, which has been elusive [20]. Proxy measures of adherence for microbicides specifically, and for PrEP more generally, have ranged from self-reported counts of use/non-use to measurement of drug concentration in the blood and vaginal fluids. All proxy measures have strengths and weaknesses [21]. For example, in the case of vaginal rings, the wide intra-individual variability in drug blood levels during use creates challenges for accurately assessing how long an ARV-based ring has been inserted or if it has been removed or expelled and reinserted [22]. Efforts are underway to identify biomarkers of adherence, for example, measurement of residual drug in rings, the quantification of biofilm on the surface of vaginal rings to assess duration of insertion and assessment of plasma drug levels aggregated by site. Nonetheless, adherence measurement remains dependent, at least in part, on self-report, which is known to be affected by social desirability biases and cognitive limits on recall [23, 24].

No single approach to measuring ring adherence will be adequate to provide the level of detail needed to 1) monitor adherence in real time to target participants for focused discussions around product use, 2) allow for statistical inferences about effectiveness while controlling for adherence and 3) inform planning for adherence assessments during programmatic implementation of effective products. Increasingly, researchers are advocating for use of several adherence measures that can be triangulated [25, 26].

While considerable innovation is emerging to provide adherence support and develop more objective measures, approaches have been largely specific to a particular study or study-site. Furthermore, the adherence support procedures used to date in microbicide or PrEP trials have been derived from theory and practice established mainly in the treatment context, with no evaluative piloting for the experimental prevention research context. In the CAPRISA 004, iPrEX and VOICE trials adherence support procedures underwent modification after trials were well underway [27, 28]. Empirical data on the fidelity of research teams to adherence support guidelines is limited, further undermining efforts to understand the effectiveness of a given adherence support approach. Biometric measures such as drug levels have similarly undergone continual refinement in the context of the very trials where those measures have been used to evaluate adherence. Innovative approaches sometimes have difficulty getting traction in clinical trials, due to concerns about their impact on trial implementation in the absence of pilot data.

## Discussion

### Developing an interdisciplinary framework

**Figure 1:**
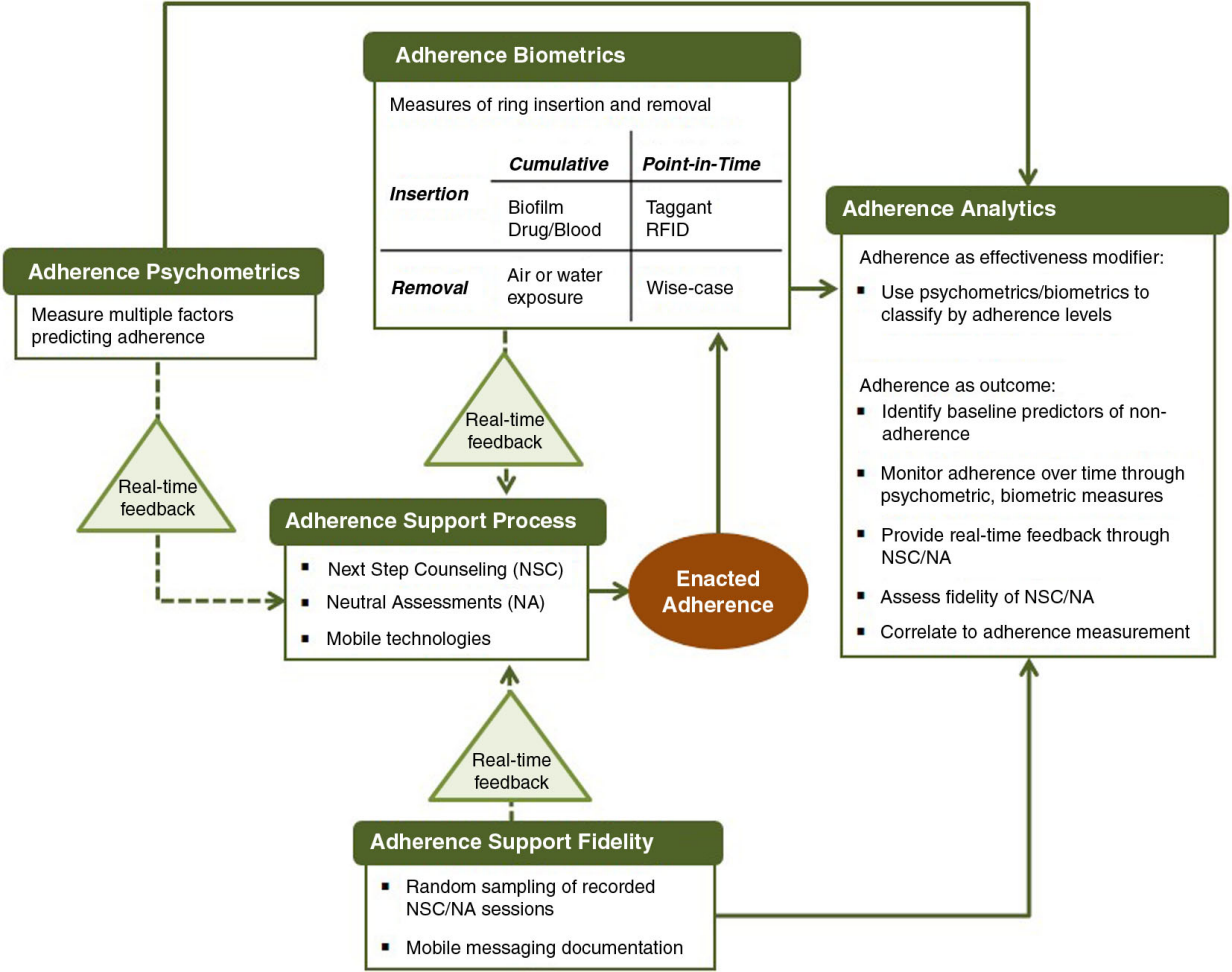
An integrated framework for adherence support and measurement in clinical trials of ARV-based vaginal rings for HIV prevention.

An interdisciplinary framework is needed to move past the current ad hoc approach and integrate the various dimensions of adherence assessments without burdening or detracting from on-going product development or trial implementation. Figure 1 describes the key components of such a framework for understanding, measuring and supporting ring adherence. This framework highlights the dual use of adherence measurement (i.e. to provide feedback during trial implementation and to inform interpretation of trial findings) and underscores the complex pathways that connect measurement, adherence support and enacted adherence behaviour.

### Adherence support process

Secondary analysis of data from the CAPRISA 004 trial suggests that use of a theory-driven approach to support adherence to product use instructions can be effective. Midway through the CAPRISA 004 trial the investigators revised their adherence support approach to a counselling package based on motivational interviewing, referred to as Adherence Support Program (ASP). Findings indicate that implementation of ASP was associated with an increase in the proportion of women reporting high adherence, an increase in detectable tenofovir levels and an increase in the estimate of product effectiveness [29]. Similarly, the Uganda Partners PrEP site conducted an ancillary adherence counselling intervention study with a subset of participants. The intervention was adapted from an evidence-based HIV treatment adherence intervention called Lifesteps [30] that included cognitive-behavioural, motivational interviewing, and problem-solving techniques and significantly increased adherence as measured by unannounced pill counts [31].

In the iPrEX PrEP trial among MSM, which also demonstrated product effectiveness, a different but somewhat related approach was developed called next step counselling (NSC). NSC was designed to promote open discussions about product use and to build motivation and positive regard for the use of an unproven product (or potentially a placebo product) within a clinical trial [32]. In conjunction with NSC, a neutral assessment (NA) approach was also implemented where rates of product use were collected by non-counselling team members who were trained to intentionally remove judgment and negative consequences (e.g. extensive follow-up, probing or additional procedures) for reporting product non-use. The combined NSC/NA approach subsequently formed the basis for adherence support procedures called VASP (VOICE adherence strengthening program) used in the latter half of the VOICE trial. Neither trial had a comparison arm by which to evaluate the combined approach. However, unlike the CAPRISA 004 and Uganda Partners PrEP findings, mid-trial adoption of VASP did not appear to increase adherence when individual adherence was examined pre- and post-VASP implementation. A NSC/NA type of approach was used in the early part of FACTS 001 (an on-going follow-on phase 3 trial to replicate CAPRISA 004), which was subsequently switched to using the ASP approach. The IPM and MTN trials currently assessing IPM's 28-day dapivirine vaginal ring draw on a variety of adherence support strategies. The MTN study uses a VASP-style approach, called Adherence Counseling Education (ACE) and a number of small group-focused discussions and activities. Common elements across all of the ARV-based trials described above include aspects of motivational interviewing and client centred models originally developed outside the research context; some also include use of product returns, self-report or biomarker (drug level) measures of product non-use to target adherence counselling with participants.

While current efforts to address the adherence support challenges in real-time with on-going clinical trials are theory-based and draw on behavioural science methods, none of the approaches used in blinded biomedical randomized controlled trials of HIV prevention have been evaluated rigorously for that context. The motivational interviewing and client-centred counselling approaches used in these trials were derived from models largely developed with U.S. populations and focused on counselling intended to engage clients in the dynamics of change, that is, moving from a behaviour or set of behaviours that are recognized as costly/unhealthy to ones that are beneficial/healthy [33]. Counsellors sought to promote investment in change, avoiding overt instruction or persuasion about what clients “should” do. Instead, counsellors respected clients’ reasons *not* to change, provided a safe place for full expression of this resistance to change, and facilitated genuine development of an appreciation and desire for an alternative to current behaviour that maximized values and perceived benefits from a client's perspective.

The reality of clinical trial research limits implementation of full motivational interviewing principles. For example, while participants in randomized controlled trials have autonomy with regard to deciding whether to enrol or whether to comply with all study requirements, they lack autonomy to actively choose the intervention to be administered – a component of choice that is core to the MI approach. Similarly, given the critical importance of protocol compliance for successful trial implementation, it is unlikely that research staff can maintain genuine appreciation for a participant's right not to adopt the trial-desired behaviour. Finally, the primary motivational basis behind MI is undermined by the potential lack of a direct (or any) health benefit accruing to the participant as a result of adopting the clinically-desired adherence behaviour.

In contrast to motivational interviewing-based approaches, compliance-oriented approaches rely on overt persuasion, use of external reinforcements or the threat of negative consequences to encourage clients to adopt a behaviour judged by an external expert to be “better for you.” However, the use of pure persuasion can be alienating and could create negative feelings towards product use and trial participation, which could further deteriorate motivation to report on product non-use in the clinical research setting. The use of motivational interviewing-based engagement with participants, with its valuing of genuine discourse and attempt to balance power between researchers and participants is arguably a better fit with the participatory kinds of engagement reflective of current standards for HIV prevention research practice [34].

Effective support for study product use in blinded HIV prevention trials requires a theory-based, informed approach that meets the specific needs of both trial participants and the trial itself. Such a *participant-centred* approach would leverage respect for autonomy, as classically defined for human research protections [35, 36], while opening a dialog to identify, develop and foster personal investment in the objectives of the prevention trial. It would acknowledge, and then work with the uncertainty of a direct health benefit and the unknown health risks accruing to the participant as a result of adherence in the experimental context of a prevention trial. It would also explicitly recognize the cultural and social dynamics that enter into a person's motivations to enrol in a trial, continue active engagement, take up product use as assigned and adhere to study requirements as requested. Unique to a participant-centred approach, the intervention focus would be the participants’ motivation towards and skills in achieving full engagement with the research goals of the study rather than specifically on product use, per se. Persistent, as-recommended product use would be one of several desired outcomes of personal engagement in the study.

### Adherence support fidelity

Anecdotal evidence suggests that fidelity to adherence support protocols by trial staff may be a significant but unmeasured factor in participant adherence to instructions for product use. The importance of fidelity was underscored in a recent assessment of MI to support ARV treatment adherence in South Africa [37]. Yet fidelity measures are rarely included in HIV prevention trials. Considerable attention has been placed on the failure of women to follow product use instructions in the FEM-PrEP and VOICE trials but very little has been focused on whether those providing the instructions and adherence support were doing so in compliance with the standards and procedures developed for the trials. In the absence of fidelity measures it is impossible to determine the relative contributions of participant motivation, adherence support system design and provider implementation. An effective adherence support process will not ensure adequate levels of adherence if it is poorly implemented.

### Adherence psychometrics

Despite shortcomings, the assessment of self-reported adherence remains a standard component of trials because it is inexpensive, relatively non-invasive and allows for immediate feedback [21]. Methods to improve validity and reliability of self-reported adherence measures have largely focused on mode of data collection (e.g. use of ACASI). However, there has been a lack of support for development of psychometric measures similar to those long used to assess treatment non-compliance [38, 39]. Psychometrically validated scales are composed of multiple items in the form of questions or statements that, when combined, measure a more complex concept that may not be directly observable [40–42]. As such, they may better assess the multiple factors likely to contribute to non-adherence.

Drawing on behavioural theory and existing social and behavioural research on acceptability and adherence, it should be feasible to identify a multidimensional set of items that assess adherence to products in the HIV prevention pipeline. The process for developing such psychometric measures is fairly straightforward. The first step is to identify relevant conceptual domains that influence ring adherence, developing specific items that adequately represent each domain. Both the published literature and unpublished qualitative data may be consulted to determine potential domains, such as product-related attitudes (i.e. perceived benefits and barriers to ring use), motivations for product use within a clinical trial setting and perceived self-efficacy to use a ring as instructed, as well as barriers potentially associated with non-adherence (i.e. low levels of knowledge, social stigma associated with product use, male partner concerns, domestic violence, lack of mobility.) Content validity – the degree to which the domains and draft items represent the underlying dimensions of vaginal ring adherence behaviour within a clinical trial context – would be assessed in part through review and feedback by a panel of behavioural and social researchers, cultural experts and/or clinicians with expertise in HIV prevention adherence. Both the content, sequence and wording of draft items, as well as the overall framing and instructions for scale administration, must then be evaluated in a small number of culturally diverse sites, using standard cognitive interviewing techniques and/or focus group discussions, to verify that the draft measure includes relevant content and that individual items are salient, well understood and easily rated by participants similar to those who might take part in vaginal ring trials.

After the draft scale items are refined, they are incorporated into a survey that also includes measures of behaviours or attitudes that are hypothesized to predict or affect adherence directly or indirectly. The survey is administered to women in one or more cultural settings who are current or former trial product users. Exploratory and confirmatory factor analysis techniques are applied to determine the final structure and content of the adherence scale, and the theoretical associations between adherence as measured by the scale and other factors/variables are explored. Once the psychometric properties of the scale measure is confirmed, opportunities to assess the predictive value of the adherence scale can then be sought, for example, by examining how well the scale score correlates with biometric indicators of product use.

A validated scale of vaginal ring adherence would potentially have multiple applications within the context of HIV prevention clinical research. It could support the trial's evaluation of vaginal ring efficacy by providing complementary evidence on adherence, particularly when biomarker adherence data may be missing or incomplete for some trial participants. The use of a composite biomarker-plus-adherence scale approach was shown to correlate better with viral load than use of biomarker data alone in one study on HAART effectiveness [43]. Additionally, given the multidimensional composition of a vaginal ring adherence scale, it could alert staff to a participant's potential issues around adherence at baseline (if, for example, commitment to correct and continuous product use ranked low among an individual's motivations for trial participation) and/or provide real-time feedback on barriers or facilitators of use to adherence support staff over the course of the trial. While the process of developing adherence psychometrics requires an upfront investment of time and resources, once a scale is validated it is a cost-effective tool to adapt and implement across the applications described above.

### Adherence biometrics

There has been considerable attention given to the development of biomarkers and biometric measures that rely less on verbal self-report by participants and provide more direct measures of events or adherence. Examples include electronic devices such as MemsCap and WisePill devices for pill storage and Wisebag for microbicide applicator storage, which allow electronic tracking of when the storage devices are opened, ostensibly for use (or, in the case of rings, for storage) of the product [44, 45]. Some biometric approaches may indicate a point-in-time when the product is used, while other approaches may indicate more cumulative use patterns. For example, two approaches currently under investigation with the potential to measure varying durations of ring use include quantification of ring biofilm (which should increase with duration of insertion) and of residual drug (which should decrease with duration of insertion). Other biometric methods have the potential to indicate ring insertion at a given point in time. One such approach is the adaptation of an esther taggant previously tested in vaginal gel to ring technologies [46]. Another approach with some potential is the use of an RFID tag that would allow random non-intrusive scanning for ring insertion. Rather than measuring ring insertion, some biometric approaches might indicate current or cumulative ring removal. For example, it might be possible to develop a cumulative measure of ring exposure to air that could indicate the proportion of time the ring was removed, or exposure to water that might indicate removal and washing.

### Adherence analytics

To address vaginal ring adherence in the comprehensive way described above, we need to consider the analytical integration of the various approaches. As illustrated in Figure 1, analytic findings from each framework component may inform other components as well as ensuring that the best science is used within each component.

Individual elements of comprehensive adherence support processes should minimally be pretested through use of cognitive interviewing techniques and piloted using comprehension assessments on the part of participants and fidelity assessments with regard to implementation by research staff prior to their use in effectiveness trials. Experimental or quasi-experimental assessments of the comparative effectiveness of different adherence support system designs would be ideal. Formative research to develop adherence support can also inform development of the psychometric measures and determine acceptability of various biometric measurement approaches in specific cultural contexts.

Examples of fidelity measures that should be included in ARV-based HIV prevention trials include the quality and extent of staff training in the method, quality and consistency in implementation, organizational support for implementation, documentation of practices and adaptation to local and emergent needs. Use of mobile phones or other technology may also be helpful in monitoring fidelity as well as adherence on the part of participants. Here again, formative research findings will be critical for developing and adapting measures to specific trials and contexts.

Points of potential interface between psychometric and biometric measurement and adherence support should be identified and consciously and overtly considered when designing measures, support strategies and guidelines for administering each.

## Conclusions

After more than two decades of biomedical HIV prevention effectiveness trials, it is now clear that randomization, blinding and use of placebo controls cannot mitigate any behavioural or sociocultural context-specific issues that might arise during the course of a trial. Rather, behaviours and contexts that drive adherence are at issue for evaluating effectiveness. Increasingly, researchers are advocating for use of several adherence measures that can be triangulated [19, 38]. Coordinated research is needed along three pathways to generate complementary measures that can then be triangulated for a comprehensive picture: 1) psychometrics to improve self-report measures reflective of behaviour; 2) electronic dispensing or storage options to capture events corresponding to intended product use or non-use; and 3) biometrics to capture indications of product ingestion or insertion, as well as frequency or duration of same. Better measurement of adherence will not, by itself, ensure that future effectiveness trials will be able to address the most basic question: does the product prevent HIV transmission? The challenges to adherence measurement must be addressed as one component of a more integrated system that has as its central focus adherence as a context-driven behaviour.
